# Evaluating Spaces for Older Adults Experiencing Homelessness: Findings From an Environmental Audit

**DOI:** 10.1093/geroni/igab046.1130

**Published:** 2021-12-17

**Authors:** Hannah Brais, Émilie Cormier, Diandra Serrano, Atiya Mahmood, Tamara Sussman, Valérie Bourgeois-Guerin

**Affiliations:** 1 Old Brewery Mission, Old Brewery Mission, Quebec, Canada; 2 Université du Québec à Montréal, Montreal, Quebec, Canada; 3 McGill University, Montreal, Quebec, Canada; 4 Simon Fraser University, Vancouver, British Columbia, Canada

## Abstract

Homeless populations require spaces and services that take into account their life trajectories. The Aging in the Right Place - Environmental Checklist (AIRP-ENV) is an environmental audit tool developed by our team to evaluate the accessibility and overall design features of housing targeted for aging individuals experiencing homelessness. Researchers in Vancouver, Calgary and Montreal employed this tool in 2021 to evaluate environmental features in selected promising practices to identify built environment factors that promote aging in the right place. Preliminary findings reveal the following themes across sites: access to communal and recreational spaces encourage social inclusion and meaningful recreation opportunities; barrier-free built environment features foster independence and safety; and access to services and amenities encourage community mobility. Findings demonstrate a need to employ a broader evaluative lens that incorporates psycho-social factors to gain a nuanced understanding of aging in the right place for older adults who have experienced homelessness.

